# COI Haplotyping and Comparative Microbiomics of the Peach Fruit Fly, an Emerging Pest of Egyptian Olive Orchards

**DOI:** 10.3390/biology12010027

**Published:** 2022-12-23

**Authors:** Mona Awad, Haifa Ben Gharsa, Omnia Abdullah ElKraly, Andreas Leclerque, Sherif M. Elnagdy

**Affiliations:** 1 Department of Economic Entomology and Pesticides, Faculty of Agriculture, Cairo University, Giza 12613, Egypt; 2 Department of Biology, Technische Universität Darmstadt, 64287 Darmstadt, Germany; 3 Bioinsecticides Production Unit, Plant Protection Research Institute, Agriculture Research Center, Ministry of Agriculture, Giza 13611, Egypt; 4 Department of Botany and Microbiology, Faculty of Science, Cairo University, Giza 12613, Egypt

**Keywords:** *Bactrocera zonata*, olive pest, invasive pest, multilocus sequence analysis (MLSA), symbiont transfer, *Erwinia*, *Providencia*, integrated pest management (IPM), *Bactrocera oleae*, *Ceratitis capitata*

## Abstract

**Simple Summary:**

The peach fruit fly is an economically relevant agricultural insect pest infesting many types of fruit and vegetables. It originated from the Indian subcontinent and was spread across the Middle East to Egypt and Sudan. Due to changing climatic conditions, its further spread in the Mediterranean region is highly probable. The present study demonstrated that the peach fruit fly has adapted to olives as a new host plant. This poses a potentially serious threat to olive cultivation in Northern African and Southern European countries. The present study used molecular genetic methods to investigate the diversity of peach fruit fly populations from Egyptian olive orchards in order to understand if adaptation to the new host plant occurred once or several times. Moreover, as it is well known that fruit flies carry symbiotic bacteria, termed “bacterial microbiome”, that help them to adapt to changing environmental conditions, the present study has compared the bacterial microbiome of peach fruit flies developing in olives and in other fruits. Several changes in the microbiome composition were identified. This knowledge can help to understand how similar adaptations happen and to develop agents or strategies for biological control of the peach fruit fly.

**Abstract:**

The peach fruit fly, *Bactrocera zonata* (Tephritidae), is economically relevant as a highly polyphagous pest infesting over 50 host plants including commercial fruit and horticultural crops. As an invasive species, *B. zonata* was firmly established in Egypt and holds potential to spread further across the Mediterranean basin. The present study demonstrated that the peach fruit fly was found multiplying in olive orchards at two distant locations in Egypt. This is the first report of *B. zonata* developing in olives. COI barcoding has revealed evidence for high diversity across these peach fruit fly populations. These data are consistent with multiple rather than a single event leading to both peach fruit fly invasion to Egypt and its adaptation to olive. Comparative microbiomics data for *B. zonata* developing on different host plants were indicative for microbiome dynamics being involved in the adaptation to olive as a new niche with a potential adaptive role for *Erwinia* or *Providencia* bacteria. The possibility of symbiont transfer from the olive fruit fly to the peach fruit fly is discussed. Potentially host switch relevant bacterial symbionts might be preferred targets of symbiosis disruption strategies for integrated pest management or biological control of *B. zonata*.

## 1. Introduction

The olive tree (*Olea europaea* L.) is a perennial crop receiving increasing attention worldwide [1], with Egypt being one of the largest olive producing countries. Its cultivated area has expanded in the past decade, from 49,000 ha in 2010 to currently 65,000 ha, and more than 29,000 Egyptians are employed in the olive industry [2]. The olive tree is exposed to many insect pests that affect both crop quality and quantity. The olive fruit fly, *Bactrocera oleae* (Rossi), is among the most economically relevant olive pests surveyed in Egypt [3]. Moreover, the Mediterranean fruit fly, *Ceratitis capitata* (Wiedemann), is often found feeding on ripe olives [4].

Due to accelerating climate change, invasive insect species are of increasing environmental and economic significance throughout the world [5]. Several fruit flies of the family Tephritidae—including the peach fruit fly, *Bactrocera zonata* (Saunders)—are considered important quarantine insect species in many countries [6,7]. *B. zonata* is highly polyphagous, attacking over 50 host plants including commercial fruits such as peach, apricot, guava, mango, and fig as well as horticultural crops such as tomato, eggplant, and potato [8,9]. However, olives have not been reported to date to be infested by *B. zonata* [8]. Spreading westward from South-East Asia, the pest was established in the Middle East and North-East Africa with important populations in Egypt and Sudan. Extensive modeling identified the coastal areas of the Mediterranean basin as potential locations for the climate change-driven proliferation of *B. zonata* [7,10,11,12]. Similar proliferation routes have been reported previously for other invasive tephritid species such as the oriental fruit fly, *Bactrocera dorsalis* (Hendel) [13]. The peach fruit fly has been intercepted several times since 2010 in locations as far North as Central France and Austria [8] and has been categorized in the A1 list of pests recommended by EPPO to be regulated as quarantine pests [14]. In Egypt, *B. zonata* was first described in 1993 [15,16] and has spread over most governorates comprising the Northern parts of the country that are characterized by a Mediterranean rather than tropical climate [10], where it competes efficiently with *C. capitata* [17]. The annual losses caused by the peach fruit fly to Egyptian fruit growers were estimated to be about 190 million Euro [8,18].

Systematically, the fruit fly genus *Bactrocera* belongs to the tephritid tribe Dacini, with *Dacus* and *Zeugodacus* being the further main genera in this taxon. The morphology-based taxonomy of the tribe was subject to substantial modifications (for review see: [19]). The introduction of molecular markers, in particular of the mitochondrial gene encoding cytochrome c oxidase subunit 1 (COI), has made phylogenies more consistent [20,21]. In addition, a multilocus sequence analysis (MLSA) scheme comprising up to six nuclear protein-encoding genes was developed for fruit fly phylogenetics [19], and COI haplotyping was evaluated as a genetic tool employed to track the geographic origin of invasive fruit fly species [22] including *B. zonata* [23].

Tephritid fruit flies commonly associate with free-living, mostly pectinolytic enterobacteria as, e.g., *Enterobacter, Klebsiella,* or *Pantoea* [24,25,26] without establishing stable or obligatory symbiotic relationships, and bacterial microbiome compositions are highly variable at both the fruit fly species, population, and developmental stage levels [26,27]. A notable exception is the olive fruit fly [28]. Olives are, during a major part of the ripening process, a hostile and nutritionally scarce environment for larval development. This is due to a range of secondary metabolites—mainly phenolic plant glycosides, e.g., oleuropein—that constitute a chemical plant defense against both feeding insects and rot-inducing bacteria and fungi [26]. The olive fruit fly has adapted to these unfavorable conditions, forming a stable association with a single bacterial symbiont, *Candidatus* Erwinia dacicola [29,30], that presumably detoxifies its diet and supplements it with amino acids and vitamins [31,32,33].

The present study constitutes the first report of *B. zonata* developing in olive groves. COI haplotyping was used to estimate the diversity within peach fruit fly populations from Egyptian olive orchards and to explore if the observed host switch may have been due to a single or multiple adaptation events. Moreover, comparative microbiomics based on peach fruit flies from different host plants was employed to assess if the reported host switch may have been correlated with changes in the microbiome compositions. Bacteria of the genera *Erwinia* and *Providencia* were prominent in the microbiome of peach fruit flies from olives but not from other host plants. Implications of these findings for microbiome dynamics and integrated pest management strategies are discussed.

## 2. Materials and Methods

### 2.1. Sampling and Morphological Identification of Fruit Flies

**Figure 1:**
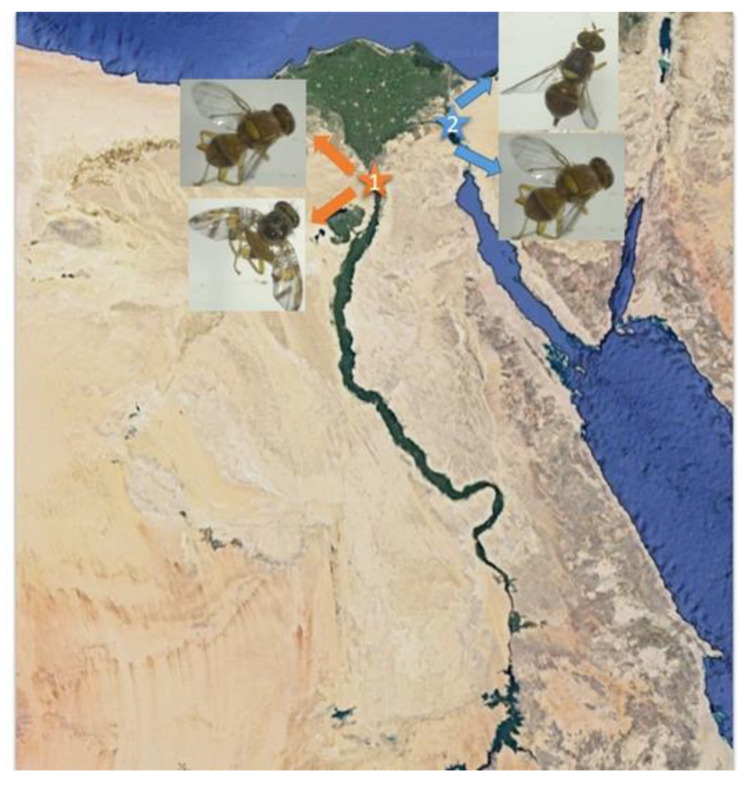
Sampling locations: (1) Giza, (2) Ismailia.

Infested fruits (guava, apple, fig, pear, mango, and olive) were collected between September and December 2019 from two different locations each in Giza and Ismailia governorates (Figure 1, Table 1). Fallen fruits were collected in mesh bags labeled with the type of fruit, date, and place of collection and kept in a cool place until processed at the laboratory. Fruits were placed in plastic trays on top of a layer of sterilized sand (5 cm) to provide a pupation site for the larvae exiting the fruit. Trays were incubated under rearing room conditions (24 ± 2 °C, 60 ± 10% RH, and 12:12 h L:D photoperiod) for up to 25 days. Mature larvae, pupae, and emerged adults were collected and transferred to clean, sterile 1.5 mL microcentrifuge tubes. Samples were stored immediately in ethanol at −20 °C until further analysis.

The morphological characteristics of fruit flies were identified using an Optika Stereozoom Microscope (SZM series) to which a toupcam digital camera (LCMOS) was attached to take fruit fly pictures. Specimens of adult fruit flies were identified based on the following morphological characters [34,35,36]. For the three species in question, adult females were distinguished from males by means of the ovipositor at the end of abdomen.

*B. zonata*

- Thorax/Scutum: red brown scutum with lateral yellow vittae (stripes) down each side, in posterior part shorter (posterior 2/3)
- Abdomen: pair of dark marks on tergite III
- Wings: lack of a complete costal band, reduced to an isolated apical spot

*B. oleae*

- Thorax/Scutum: black scutum, lateral and medial vittae absent
- Abdomen: terga with dark anterolateral corners
- Wings: costal band absent and apical spot (small spot around the apex of R4 + 5)

*C. capitata*

- Thorax/Scutum: yellowish scutum with numerous black areas in a characteristic pattern
- Wings: wings are relatively broad in comparison to length, cloudy yellow, with three brown bands separated from each other and small dark irregular streaks in the proximal half of the wing

### 2.2. DNA Extraction and PCR Amplification

For DNA extraction, whole individual insects, or groups of up to five insects from the same environmental sample, that had been morphologically identified and stored in ethanol, were frozen in liquid nitrogen and ground manually with a pestle in lysis buffer. DNA was extracted from the homogenized sample using the DNeasy Blood & Tissue kit insect protocol as provided by the manufacturer (Qiagen, Hilden, Germany) including proteinase K digestion and a 3 h treatment with 20 mg/mL lysozyme at 37 °C. Genomic DNA was eluted in 10 mM TrisCl (pH 8.5). DNA quality and quantity were controlled by horizontal agarose gel electrophoresis and photometrically using a NanoDrop NT-1000 UV spectrophotometer.

Partial sequences of the following marker genes were amplified from DNA samples extracted from individual insects using standard Taq DNA polymerase (New England Biolabs) with the PCR primers and parameters as specified in Table 2:

- the COI gene encoding cytochrome c oxidase I gene (primer pair LCO1490-mod/HCO2198-mod),
- the *ef1a* gene encoding translation elongation factor 1 alpha (M46-1/M4rc),
- the CAD1 region of the gene encoding the trifunctional carbamoyl-phosphate synthetase 2—aspartate transcarbamylase—dihydroorotase protein CAD (CAD-Bd-F/R),
- the *per* gene encoding the PERIOD protein involved in circadian rhythm regulation (Per2612F/Per3105R).

The generalized PCR protocol consisted of one initial denaturation step of 95 °C for 2 min, 35 cycles across three steps of 45 s at 95 °C (denaturation), 45 s at 52 °C (annealing) and 60 s at 68 °C (elongation), followed by a 5 min final elongation step. PCR product size was controlled by agarose gel electrophoresis, and PCR products were purified using the Qiaquick PCR purification kit (Qiagen). Sanger sequencing of PCR products was performed by StarSEQ GmbH (Mainz, Germany) using PCR and additional sequencing primers (Table 2). Raw sequence data were combined into consensus sequences using version 6 of the MEGA software package [37].

### 2.3. COI Haplotype Analysis

For the determination of COI haplotypes of fruit flies, two independent PCR reactions using primers LCO1490-mod and HC02198-mod (see Supplementary Data) were performed, purified, and sequenced from each fruit fly sample to exclude statistical errors due to nucleotide mis-incorporations or sequencing mistakes, and two independently obtained consensus sequences were generated for each sample from raw data. In case of deviations, the performance of a third PCR reaction from the respective sample was foreseen. Moreover, partial sequences were amplified using primer pairs LCO1490-mod/ Dac-COI-r and Dac-COI-f/HC02198-mod, purified and sequenced to check for the unsolicited amplification from nuclear-encoded pseudogenes [21]. COI consensus sequences were aligned and analyzed in MEGA for the occurrence of Single Nucleotide Polymorphisms (SNP). Identified COI haplotypes were compared to the Barcode of Life Database (BOLD) [38] using the assigned species level search option (http://www.barcodinglife.org, accessed on 1 November 2022).

### 2.4. Phylogenetic Reconstruction

For the reconstruction of fruit fly phylogenies, sets of reference sequences were generated as subsets of the sequence data published by Krosch et al. [21] for the COI and San José et al. [19,22] for the MLSA phylogeny (Table S1). For cumulative analysis, the three MLSA marker genes were concatenated into one meta-gene sequence and analyzed as such. Marker alignment and phylogenetic reconstruction were performed in MEGA. Phylogenies were reconstructed using a p-distance matrix-based Neighbor Joining (NJ) method with tree topology confidence limits being explored in non-parametric bootstrap analyses over 1000 pseudo-replicates.

### 2.5. Microbiome Sequencing

For microbiome analysis, DNA was extracted from up to five pooled female adult peach fruit flies from different host plants and geographic locations (Table 3). Microbiome sequencing was performed by StarSEQ GmbH, Mainz, Germany. For each sample, the variable V3-V4 region of bacterial 16S rRNA genes was amplified using primers 341f and 806bR, giving rise to amplicons comprising app. 550 bp in length. The standard Illumina 16S library preparation protocol was followed, and amplicons were double-indexed using the Nextera^®^ XT DNA Index Kit (Illumina, San Diego, CA, USA). Indexed paired-end libraries were pooled in equimolar amounts and sequenced on an Illumina MiSeq platform at 2 × 300 bp reads using reagent V3. Quality trimmed and demultiplexed sequence data were analyzed along the MiSeq Reporter pipeline, applying a 97% similarity threshold value for OTU cluster assignment and a minimal cut-off value of 0.005% of all analyzed reads for OTUs to be considered for downstream analyses.

## 3. Results

### 3.1. Sampling and Morphological Identification of Fruit Flies

**Figure 2:**
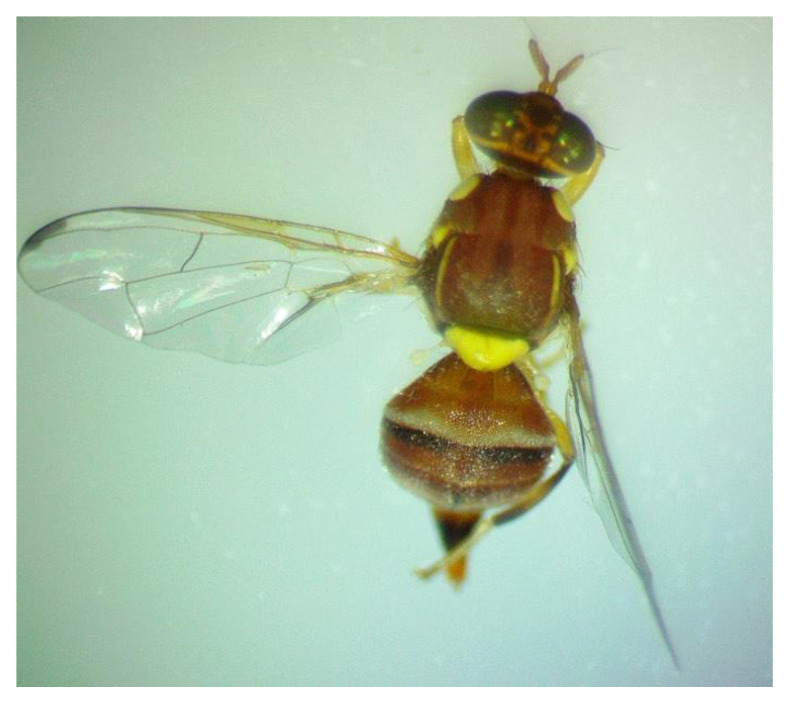
Dorsal and wing view of adult peach fruit fly, *B. zonata* (Saunders), sampled from olive at Giza-1 site.

Adult male and female fruit flies obtained from plant material sampled from Giza or Ismailia sites were taxonomically identified as peach fruit fly, *B. zonata* (Saunders)*,* Mediterranean fruit fly, *C. capitata* (Wiedemann), or olive fruit fly, *B. oleae* (Rossi), respectively, as indicated in Table 3. A picture of a peach fruit fly adult sampled at Giza-1 is presented in Figure 2.

### 3.2. Molecular Taxonomy of Fruit Flies

For the molecular taxonomic identification of flies, DNA was extracted from six individual adults each from Giza and Ismailia that had morphologically been assigned to *B. zonata*. Moreover, four flies from Giza characterized as *C. capitata* and four flies from Ismailia determined to be *B. oleae* were included in the analysis. Each group consisted of equal numbers of females and males. Samples from individual insect specimens were labeled by a three-letter code indicating the geographic origin (“G” for Giza, “I” for “Ismailia), the morphology-based taxonomic assignment (“M” for “Mediterranean fruit fly”, “O” for “olive fruit fly”, “P” for “peach fruit fly”) and gender (“M”, “F”) plus a sequential number. 

Consistent consensus sequences comprising in length 573 bp (COI), 510 bp (CAD1), 759 bp (*ef1a*) and 450 bp (*period*) were obtained for all fruit fly samples investigated except *C. capitata* sample GMF2 that did not give rise to a CAD1 PCR product. For the COI marker, two independent amplification reactions from the same sample always gave rise to pairs of identical consensus sequences. Moreover, no diverging partial sequences from potentially nuclear-encoded COI pseudo-genes were identified. COI gene and MLSA marker sequences determined were submitted to the Genbank database, respectively, under accession numbers MZ350634–MZ350653 and MZ358323–MZ358386 (Table S2).

**Figure 3:**
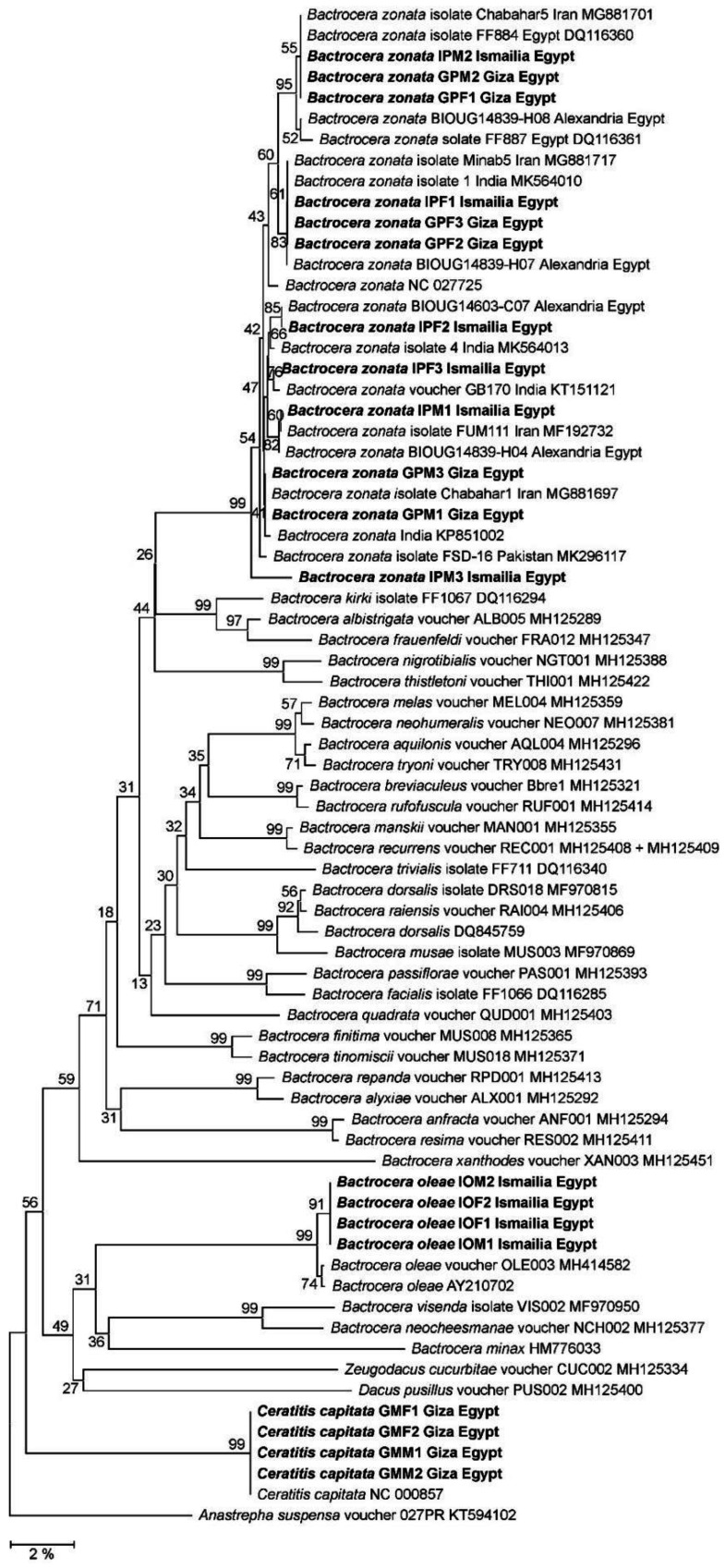
Neighbor Joining (NJ) phylogeny of fruit flies of the genus *Bactrocera* as reconstructed from mitochondrial genes encoding cytochrome c oxidase subunit 1 (COI). Terminal branches are labelled by genus, species, specimen, geographic origin designations, and Genbank accession numbers (for reference sequences). Sequences from the present study are displayed in bold type. Numbers on internal branches designate bootstrap support percentages. The size bar indicates a branch length corresponding to 2% sequence divergence. The orthologous sequence from the Caribbean fruit fly, *Anastrepha suspensa*, was used as outgroup.

COI sequence-based phylogenetic reconstruction (Figure 3) located fruit flies from Giza and Ismailia in at least 99% bootstrap-supported clades comprising *B. zonata, B. oleae,* or *C. capitata* reference sequences in perfect congruence with previous morphology-based species-level assignments. The three clades were clearly delineated from each other and from clades representing further *Bactrocera* species. Within the *B. zonata* clade, fruit flies from both sampling locations formed a highly polyphyletic group, being individually inserted into weakly bootstrap-supported sub-clades comprising *B. zonata* from geographic origins as India, Pakistan, Iran, and Egypt. There was no clustering with respect to the geographic origins of these samples at neither the country nor the sampling location levels. 

**Figure 4:**
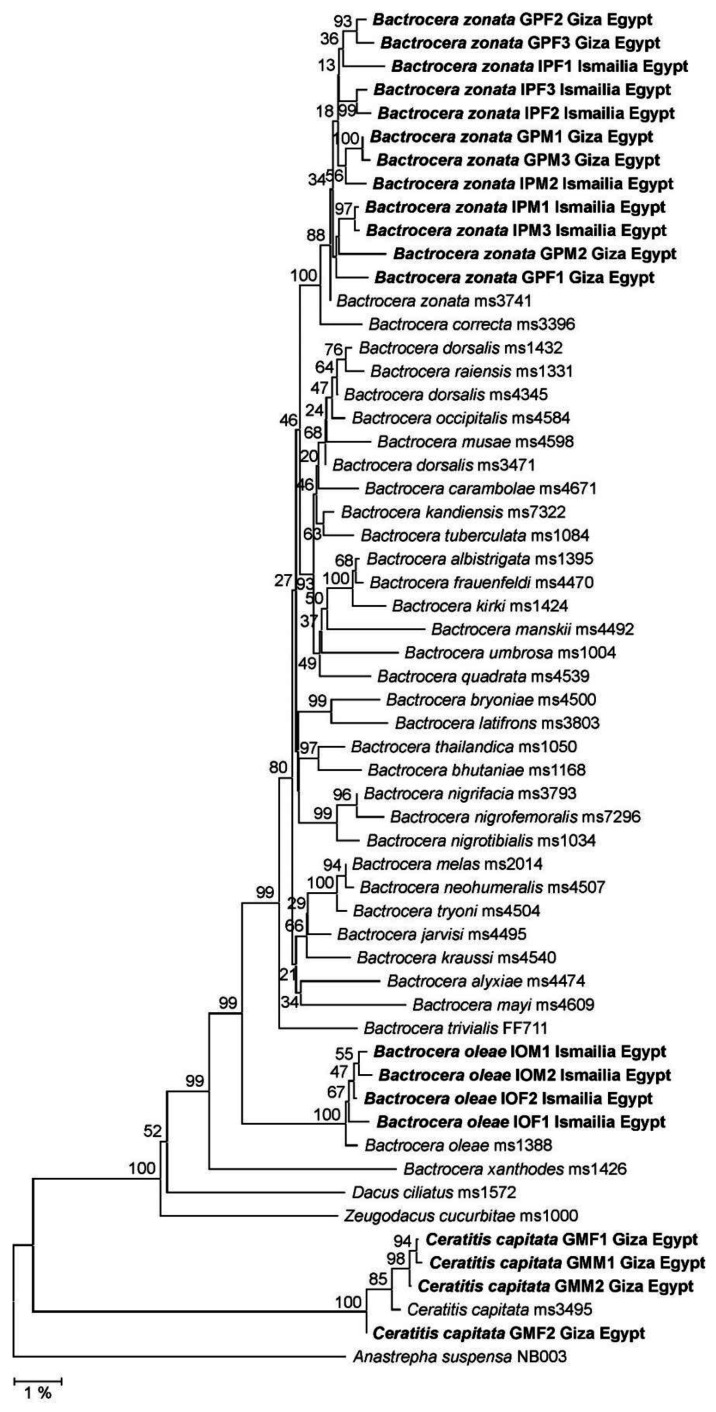
Neighbor Joining (NJ) phylogeny of fruit flies of the genus *Bactrocera* as reconstructed from a concatenation of genes CAD1, EF1A, and PERIOD. Terminal branches are labelled by genus, species, specimen, and geographic origin designations (for sequences from this study). Sequences from the present study are displayed in bold type. Numbers on internal branches indicate bootstrap support percentages. The size bar designates a branch length corresponding to 1% sequence divergence. The concatenation of orthologous sequences from the Caribbean fruit fly, *Anastrepha suspensa*, was used as outgroup.

In the phylogeny reconstructed from concatenated MLSA marker sequences (Figure 4), the corresponding *B. zonata, B. oleae,* or *C. capitata* clades received 99–100% bootstrap support. No clustering for sampling locations was supported across the *B. zonata* clade, fully in line with results from the COI phylogeny. Moreover, the analysis of obtained COI sequences in the BOLD database (Table 4) was perfectly congruent with the phylogenetic reconstruction results. Taking these fully consistent outcomes together, the four complementary identification approaches gave rise to unambiguous species-level assignments for all samples investigated.

### 3.3. Diversity of Fruit Fly Populations

Whereas all Mediterranean fruit flies from Giza and all olive fruit flies from Ismailia carried COI genes of identical nucleotide sequence, COI sequences determined for peach fruit flies from both locations were highly diverse. For the *B. zonata* COI genes, seven haplotypes termed A-G were identified (Table 4), with three (namely B, D and E) and six (A, C–G) haplotypes being found in insects originating from Giza and Ismailia, respectively. This means that each analyzed individual insect sample from Ismailia reproducibly gave rise to a unique COI haplotype. Haplotype sequence analysis identified 23 positions carrying single nucleotide polymorphisms across the amplified 573 bp COI marker sequence (Table S3). All identified SNPs caused silent nucleotide substitutions, with deduced COI amino acid sequences of all haplotypes being identical. Identified SNPs were distributed over the entire amplified COI partial sequence, with pairs of haplotypes differing mostly by multiple SNPs.

When compared to COI sequence entries of the BOLD and Genbank databases, *B. zonata* with the most closely related COI haplotypes, including exact matches for haplotypes A-E and G, were from India, Pakistan, Iran, and Egypt (Table 4, Figure 3). However, to our knowledge, *B. zonata* COI haplotype F was not previously reported.

### 3.4. The Bacterial Microbiome of B. zonata from Different Fruits

The bacterial microbiome of female peach fruit flies from olive and further fruits such as apple, guava, pear, and mango at Giza and Ismailia sampling sites was determined by 16S pyrosequencing (Table 5). In general, main microbiome components were assigned to the bacterial genera *Enterobacter* and *Klebsiella*, whereas less abundant components belonged to the genera *Erwinia, Pseudomonas, Trabulsiella, Acetobacter,* and *Gluconobacter*. The latter two bacterial genera appeared, however, to be absent from the microbiome of the *B. zonata* adults obtained from olives, whereas the relative abundance of *Pseudomonas* bacteria increased in the respective samples with the effect being particularly pronounced for the olive samples stemming from the Ismailia sampling site. Interestingly, *Providencia* and *Erwinia* bacteria were prominent components of the microbiome of the peach fruit flies from olives originating from the Ismailia, but not the Giza sampling sites.

## 4. Discussion

Using several independent methods, namely morphological characterization of adult flies, phylogenetic reconstruction from COI gene and MLSA marker sequence data, as well as comparison with the BOLD COI species database, fruit flies sampled from infested olives at Giza and Ismailia, Egypt, were conclusively assigned to the taxonomic species *B. zonata, B. oleae,* and *C. capitata*. Whereas the latter two are well-known to multiply and feed on olive, the peach fruit fly has to date not been reported as an olive pest [39]. As *B. zonata* was found developing in olives at two distant locations, the respective host switch does not appear to be a single event but is firmly established at least in Northern Egypt. In the light of the predicted proliferation potential of the peach fruit fly towards further countries North and South of the Mediterranean Sea [7], including important olive-cultivating countries such as Tunisia or Italy, economic consequences of this host switch might be highly relevant.

Despite the small number of samples available for the present study, the generated COI haplotyping results were suggestive of an increased level of biodiversity in both peach fruit fly populations. COI haplotype sequence similarities appeared to reflect the currently accepted expansion history of the peach fruit fly. *B. zonata* has probably originated on the Indian subcontinent, diversified in South-East-Asian rain forests, and later spread westward to Iran, the Arabian Peninsula, and Northern Africa [20]. In fact, the tephritid tribe Dacini was treated as a text-book example for the evolutionary “out-of-India” hypothesis [40]. Studies of *B. zonata* populations in India and Bangladesh demonstrated a very high degree of COI haplotype diversity in these supposedly native regions [23,41]. A previous study of Egyptian *B. zonata* isolates sampled from infested guava (*Psidium guajava)* fruits at five different locations across Giza governorate revealed a single identical COI haplotype for all samples [42]; however, the study was retracted later [43]. In contrast, sampling of *B. zonata* at a single location, namely the Antoniades Gardens at Alexandria, has revealed numerous COI haplotypes (sequence data published under sample-IDs BIOUG14603-XXX in the BOLD database). 

A low haplotype diversity in an invasive range in contrast to a high diversity level of the same species in its native range would be indicative for a restricted invasion by a small initial invasive population and/or a limited number of invasion events and would at least in principle enable tracking of geographic origins and invasion routes of the invasive population [20]. However, despite the statistically insufficient size of the fruit fly sample analyzed in this study, the apparently increased level of COI haplotype diversity determined in *B. zonata* specimens from Giza and Ismailia governorates was suggestive of the opposite invasion scenario of numerous independent invasion events and/or the introduction of large initial invasive populations. Correspondingly, in the COI-based phylogeny, peach fruit fly specimens sampled in Egypt were found widely distributed among *B. zonata* from countries alongside the potential invasion route, i.e., India, Pakistan, and Iran (Figure 3). A substantially identical picture has arisen from the best hits identified in the BOLD database (Table 4).

In previous studies, the main microbiome components associated with adult peach fruit flies from different environmental contexts were identified to belong to the bacterial genera *Enterobacter* and *Klebsiella* [44,45,46] with an additionally high relative abundance of *Lactococcus* in larvae [27] and of *Pseudomonas* in pupae of *B. zonata* [46]. The microbiome composition established in the present study from female adults of *B. zonata* (Table 5) showed an apparently high relative abundance of *Enterobacter* and *Klebsiella* bacteria irrespective of the host fruit. Bacteria of the genus *Lactococcus* were not identified in any of the adult flies analyzed. The apparently low to intermediate relative abundance of several further bacterial genera is in line with published data [46] concerning *Gluconobacter* and *Pseudomonas*. To our knowledge, bacteria of the genera *Acetobacter, Erwinia,* and *Trabulsiella* have not been previously described as associated with peach fruit flies. However, *Erwinia* and *Acetobacter* bacteria, more exactly *Candidatus* Erwinia dacicola [29,30] and *Acetobacter tropicalis* [47], are well-established symbionts of the olive fruit fly [28], whereas the genus *Trabulsiella* comprises gut symbionts of fungus-growing termites [48] and a component of the midgut microbiome of *Anopheles* mosquitoes [49]. 

Concerning the host plant, the comparison of microbiome compositions is—under the caveat of a sample size too small to permit meaningful statistical analysis—suggestive of peculiarities for *B. zonata* developing in and feeding on olives as opposed to a range of other fruits more typically infested by the peach fruit fly. The most notable changes apparently associated with a hypothetical host switch from other plants towards olive might be the disappearance of *Acetobacter* and *Gluconobacter* bacteria from the *B. zonata* microbiome, together with an apparent increase in the relative abundance of *Pseudomonas, Erwinia,* and *Providencia* bacteria. *Providencia* bacteria were previously identified in *Drosophila melanogaster* [50,51] and several tephritid flies as the Mexican fruit fly, *Anastrepha ludens* [52], *C. capitata* [53], and *B. oleae* [54].

The significance of the host plant for the olive fruit fly microbiome composition was addressed previously with the notion of a host plant–microbe–fly interaction [55]. It was shown that bacteria associated with the alimentary tract of fruit flies reside on the host plant as, e.g., on the olive phylloplane [56,57,58]. Volatile organic compounds emitted by these bacteria attract fruit flies towards the host plant [59]. Moreover, it was demonstrated that fruit fly-associated bacteria were introduced into the host fruit during oviposition, and that bacterial amplification in the infested fruit was an important step in microbiome establishment in developing larvae [25,60]. Therefore, growth of potential fruit fly-symbiotic bacteria on or in the host plant might be a key factor promoting the exchange of bacterial symbionts between different fruit fly species infesting the same plant.

The results of this study as displayed in Table 3 and Table 5 indicate that the relative abundance of *Erwinia* and *Providencia*, i.e., of bacteria known to be associated with the olive fruit fly, appeared high (13.2% and 30.9%, respectively) in *B. zonata* from olives co-infected with *B. oleae* (i.e., from location Ismailia-2), but low (1.5% and 0%) in *B. zonata* from olives not infected with *B. oleae* (Giza-1). Therefore, one might hypothesize that the establishment of these bacteria in *B. zonata* might be explained by host plant-mediated symbiont transfer from the olive fruit fly to the peach fruit fly. Coincidental uptake of olive fruit fly symbionts by peach fruit flies or larvae feeding on or multiplying in olive might subsequently be stabilized by selection for the symbiont-conferred ability to cope better with harsh conditions in unripe olives. For instance, *Erwinia* bacteria detoxifying phenolic plant glycosides such as oleuropein might confer similar adaptive advantages to *B. zonata* developing in unripe olives, as is the case for *B. oleae*. Whereas the metabolic contribution of *Candidatus* Erwinia dacicola appeared to be obligatory for olive fruit fly larvae developing in unripe olives, it was previously demonstrated to be facultative in ripe olives or on artificial diet [26,61] with *Candidatus* Erwinia dacicola being potentially substituted by more loosely associated bacteria as, e.g., *Enterobacter* [32,33], *Acetobacter* [47], or *Tatumella* [33].

The scenario of *B. zonata* as a highly invasive new olive pest spreading further around the Mediterranean basin holds major agricultural and economic implications. Monitoring and pest management activities targeting the peach fruit fly will have to be put in place. If *B. zonata* development in unripe olives will by the end of the day turn out to be symbiont-dependent, biological control or integrated pest management approaches targeting the fruit fly–symbiont interaction might become attractive pest management options to be seriously explored. Concerning the olive fruit fly, several respective approaches have been proposed [62,63,64,65,66], and analogous symbiosis disruption strategies might be highly solicited to protect Mediterranean olive orchards from peach fruit fly infestation.

## 5. Conclusions

The present study demonstrated that the peach fruit fly, *B. zonata*, multiplies in olives at two distant locations in Egypt. To the authors’ knowledge, this is the first report of *B. zonata* having adapted to olive as host plant. As *B. zonata*, a highly invasive species, is predicted to spread further around the Mediterranean basin, this host switch potentially represents an economically relevant threat to Mediterranean olive cultures. 

COI barcoding results obtained were suggestive of an increased diversity across both peach fruit fly populations under study. Comparative microbiomics data for *B. zonata* from different host plants is suggestive of microbiome dynamics being involved in the adaptation to olive as a new niche. In particular, *Erwinia* and *Providencia* bacteria known to be associated with the olive fruit fly, *B. oleae*, appeared prominent in the microbiome of peach fruit flies originating from olive orchards infested simultaneously by both *Bactrocera* species. This finding is suggestive of the idea of a host-mediated transfer of symbionts between pests when feeding or multiplying on the same plant. Beyond fundamental research interests, symbiont associations enabling *B. zonata* to infest olive are a potential target for the development of pest management strategies to limit the economic impact of this new invasive olive pest.

## Figures and Tables

**Table 1:** Sampling locations.

Acronym	Location	GPS Coordinates
Giza-1	Agricultural Research Center, Giza	30°01′23.8″ N 31°12′26.0″ E
Giza-2	Cairo–Alexandria Road, Wadi Food Farm, Giza	30°14′17.6″ N 30°47′14.6″ E
Ismailia-1	El Ferdan, Ismailia	30.655947° N, 32.325767° E
Ismailia-2	Suez Canal University Farm, Ismailia	30.6205° N, 32.2697° E

**Table 2:** Oligonucleotide primers used in this study.

Primer Designation	Primer Sequence	Reference
LCO1490-mod	5′-TYTCAACAAATCATAAAGATATTGG-3′	[21]
HC02198-mod	5′-TAAACTTCAGGGTGWCCAAARAATCA-3′	
Dac-COI-f	5′-GCHTTCCCHCGAATAAATAATA-3′	
Dac-COI-r	5′-GTTCAACCTGTACCVGCYCCGTTTTC-3′	
M46-1	5′-CAGGAAACGCTATGACCGAGGAAATYAARAAGGAAG-3′	[19]
M4rc	5′-TGTAAAACGACGGCCAGTACAGCVACKGTYTGYCTCATRTC-3′	
CAD-Bd-F	5′-CCGGTAAATTTTGAATGGTTC-3′	
CAD-Bd-R	5′-GCRGTKGCGAGCARYTGATG-3′	
Per2612F	5′-ATTCATGGGAAGGAGATGCC-3′	
Per3105R	5′-AABGACATGGGTTGGTACATC-3′	

**Table 3:** Fruit fly sampling and identification. Numbers in brackets in the “female adults” column indicate numbers of individuals pooled for microbiome analysis.

Geographic Origin	Sampling Date	Host Plant	Male Adults	Female Adults	Identification
Giza-1	<09/2019	Guava	10	10 (5)	*Bactrocera zonata*
Giza-1	<09/2019	Apple	10	10 (5)	*Bactrocera zonata*
Giza-1	<09/2019	Pear	5	3 (3)	*Bactrocera zonata*
Giza-1	10–11/2019	Olive	8	8 (5)	*Bactrocera zonata*
Giza-2	10–11/2019	Olive	2	3	*Ceratitis capitata*
Ismailia-1	<09/2019	Mango	0	1 (1)	*Bactrocera zonata*
Ismailia-1	<09/2019	Pear	0	1 (1)	*Bactrocera zonata*
Ismailia-2	10–11/2019	Olive	6	8	*Bactrocera zonata*
Ismailia-2	10–11/2019	Olive	4	6	*Bactrocera oleae*
Ismailia-2	12/2019	Olive	3	6 (5)	*Bactrocera zonata*

**Table 4:** Comparison of COI sequence data to the BOLD COI species database.

Sample-ID (Haplotype)	Best Hit—ID	Maximal Similarity Percentage	Minimal Similarity Percentage	Best Hit (Accession Number, Geographic Origin)
IPF3 (A)	*Bactrocera zonata*	100	99.13	MK564024, India
GPM3 (B)	*Bactrocera zonata*	100	98.95	MG881761, Iran
IPF2 (C)	*Bactrocera zonata*	100	98.78	BIOUG14603-C07, Egypt
GPF1 (D)	*Bactrocera zonata*	100	98.43	MG881762, Iran
GPF2 (E)	*Bactrocera zonata*	100	98.78	MK564010, India
IPM3 (F)	*Bactrocera zonata*	98.34	97.24	MK296117, Pakistan
IPM1 (G)	*Bactrocera zonata*	100	98.78	MF192732, Iran
GMF1	*Ceratitis capitata*	100	99.65	MT474895, Australia
IOF1	*Bactrocera oleae*	100	99.3	KY111512, Turkey

**Table 5:** Relative abundance of bacterial genera in microbiomes associated with female adults of *B. zonata* from different origins (in % of all analyzed reads).

Location	Giza-1				Ismailia-1		Ismailia-2
Fruit	Apple	Guava	Pear	Olive	Mango	Pear	Olive
***Enterobacter***	69.3	34.8	73.9	54.1	66.1	32.5	34.7
***Klebsiella***	7.7	26.8	7.7	15.8	14.6	49.0	3.6
***Acetobacter***	6.5	2.5	1.5	**0**	1.1	4.3	**0**
***Gluconobacter***	1.3	0.7	0.4	**0**	0.3	2.0	**0**
***Trabulsiella***	2.9	2.7	2.1	5.5	3.9	3.8	1.2
***Pseudomonas***	0.7	0.8	0.3	**4.6**	0	0	**8.1**
***Erwinia***	0.5	1.5	0.4	1.5	5.1	0.7	**13.2**
***Providencia***	0	0.1	0	0	0	0	**30.9**

## Data Availability

Sequence data analyzed in this study are publicly available from the Genbank database (https://www.ncbi.nlm.nih.gov) under nucleotide sequence accession numbers listed in Supplementary Tables S1 and S2 to this study. Microbiome data analyzed in this study are publicly available from the Open Science Framework (OSF) database under link: https://osf.io/kr2pz/?view_only=56a622654f5b48bebfc762851d79decf.

## Supplementary Materials

The following supporting information can be downloaded at: https://www.mdpi.com/article/10.3390/biology12010027/s1: Table S1: Description of reference specimen and Genbank accession numbers of reference sequences used for phylogenetic reconstruction. Table S2: Fruit fly samples from olive (Olea europaea) investigated in this study and Genbank accession numbers of marker sequences determined. Table S3: Single Nucleotide Polymorphisms (SNPs) identified in COI haplotypes of *B. zonata* from olive. Supplementary Data: PCR amplification of cytochrome c oxidase subunit 1 (COI) marker sequences from *Bactrocera zonata* from Egypt.
