# Supplementary material for: COI Haplotyping and Comparative Microbiomics of the Peach Fruit Fly, an Emerging Pest of Egyptian Olive Orchards
Source: Biology (Basel). 2022 Dec 23;12(1):27. doi: 10.3390/biology12010027 (PMC9855353; doi:10.3390/biology12010027)

Supplementary Material

COI haplotyping and comparative microbiomics of the peach fruit fly, an emerging pest of Egyptian olive orchards

Mona Awad, Haifa Ben Gharsa, Omnia Abdullah ElKraly, Andreas Leclerque, and Sherif M. Elnagdy

PCR amplification of cytochrome c oxidase subunit 1 (COI) marker sequences from *Bactrocera zonata* from Egypt

Templates:	Sixteen DNeasy preps from individual adult fruit flies from two locations in Egypt, sampled in 10-11/2019. For the molecular taxonomic identification of flies, DNA was extracted from six individual adults each from Giza and Ismailia that had morphologically been assigned to *Bactrocera zonata*. Moreover, four flies from Giza characterized as *Ceratitis capitata* and four flies from Ismailia determined to be *Bactrocera oleae* were included in the analysis. Each group consisted of equal numbers of females and males. Samples from individual insect specimens were labeled by a three-letter code indicating the geographic origin ("G" for Giza, "I" for "Ismailia), the morphology-based taxonomic assignment ("M" for "Mediterranean fruit fly", "O" for "olive fruit fly", "P" for "peach fruit fly") and gender ("M", "F") plus a sequential number.
PCR primer pair	LCO1490-mod / HCO2198-mod

PCR cycling parameters:

Step1	95°C	2:00		
Step2	95°C	0:45		
Step3	**52°C**	0:45		
Step4	68°C	**1:00**	-> 2	**35** cycles
Step5	68°C	5:00		
Step6	10°C	pause		

5 ul of each sample were used for electrophoresis (80 V for app. 45 min) on 1% agarose gel in 1x TAE, stained with 5ul/100ml Roti-Gelstain (in gel).

Gel 1

Order of samples (left to right):
B. oleae DNA as positive control, negative control, 1kb ladder size standard, 1st reactions IPF1, IPM1, 2nd reactions IPF1, IPM1.

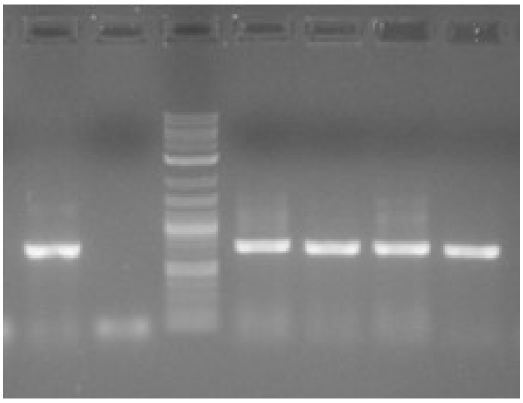

Gel 2

First row (left to right):
1st reactions GMF1, GMF2, GMM1, GMM2, GPF1, GPF2, GPF3, GPM1, GPM2, GPM3, 1kb ladder size standard, IOF1, IOF2, IOM1, IOM2

Second row (left to right):
2nd reactions GMF1, GMF2, GMM1, GMM2, 1kb ladder size standard, GPF1, GPF2, GPF3, GPM1, GPM2, GPM3, IOF1, IOF2, IOM1, IOM2

Third row (left to right):
1st reactions IPF2, IPF3, IPM2, IPM3, no template control, 1kb ladder size standard, 2nd reactions IPF2, IPF3, IPM2, IPM3

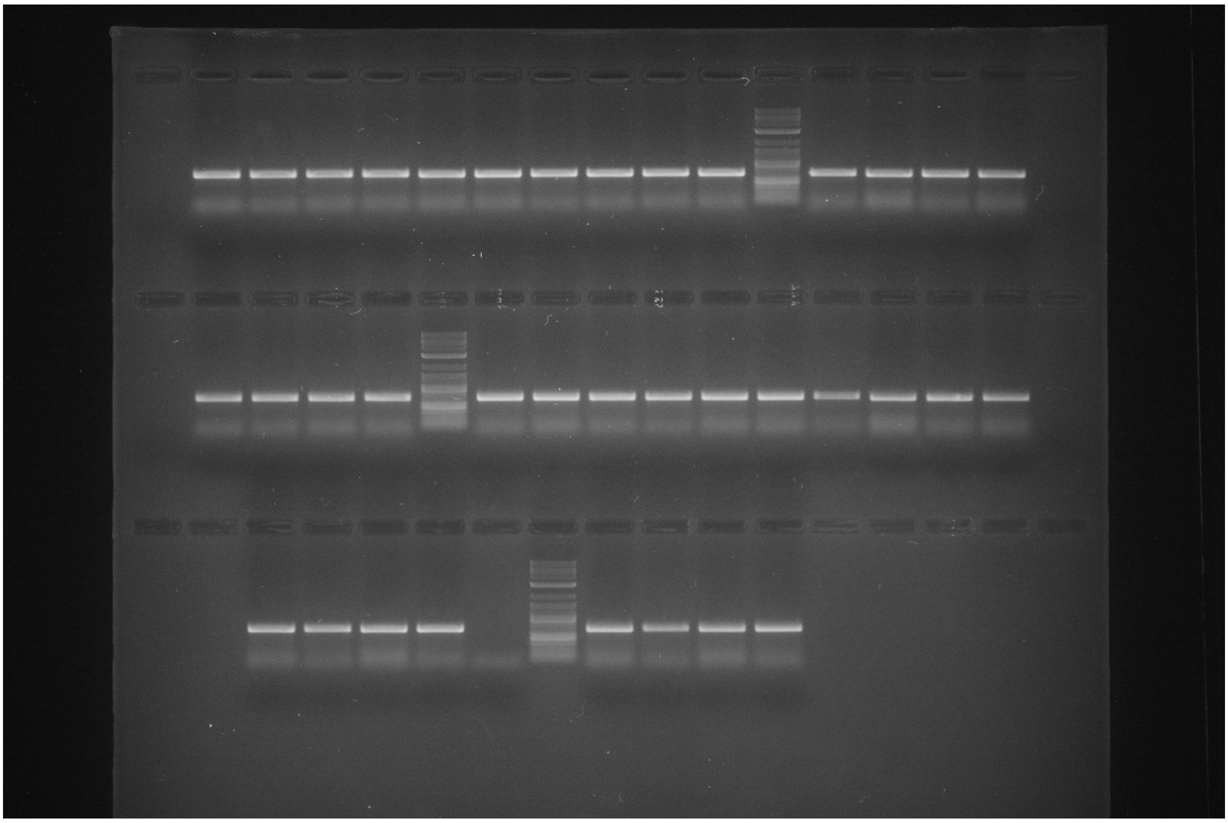

Supplement: Supplementary file 1 [file biology-12-00027-s001.zip › biology-2097061-supplementary-Date.pdf]
