# Supplementary material for: COI Haplotyping and Comparative Microbiomics of the Peach Fruit Fly, an Emerging Pest of Egyptian Olive Orchards
Source: Biology (Basel). 2022 Dec 23;12(1):27. doi: 10.3390/biology12010027 (PMC9855353; doi:10.3390/biology12010027)
Supplement: Supplementary file 1 [file biology-12-00027-s001.zip › biology-2097061-supplementary-Tables.pdf]

Table S1. Description of reference specimen and Genbank accession numbers of reference sequences used for phylogenetic reconstruction.

Genus	Species	Specimen Voucher	CAD1	EF1A	Period
Anastrepha	*suspensa*	NB003	MG683197	MG683674	MG683835
Bactrocera	*albistrigata*	ms1395	MG683054	MG683539	MG683695
Bactrocera	*alyxiae*	ms4474	MG683120	MG683607	MG683758
Bactrocera	*bancroftii*	ms4596	MG683145	MG683633	MG683784
Bactrocera	*bhutaniae*	ms1168	MG683045	MG683532	MG683688
Bactrocera	*bryoniae*	ms4500	MG683131	MG683618	MG683769
Bactrocera	*carambolae*	ms4671	MG683151	MG683640	MG683791
Bactrocera	*correcta*	ms3396	MG683083	MG683572	MG683722
Bactrocera	*dorsalis*	ms4345	MG683111	MG683598	MG683749
Bactrocera	*dorsalis (invadens)*	ms3471	MG683088	MG683577	MG683727
Bactrocera	*dorsalis (papayae)*	ms1432	MG683058	MG683544	MG683699
Bactrocera	*frauenfeldi*	ms4470	MG683119	MG683606	MG683757
Bactrocera	*jarvisi*	ms4495	MG683128	MG683615	MG683766
Bactrocera	*kandiensis*	ms7322	MG683189	MG683669	MG683829
Bactrocera	*kirki*	ms1424	MG683056	MG683542	MG683697
Bactrocera	*kraussi*	ms4540	MG683137	MG683624	MG683775
Bactrocera	*latifrons*	ms3803	MG683106	MG683593	MG683744
Bactrocera	*manskii*	ms4492	MG683126	MG683613	MG683765
Bactrocera	*mayi*	ms4609	MG683149	MG683637	MG683788
Bactrocera	*melas*	ms2014	MG683075	MG683564	MG683714
Bactrocera	*musae*	ms4598	MG683146	MG683634	MG683785
Bactrocera	*neohumeralis*	ms4507	MG683134	MG683621	MG683772
Bactrocera	*nigrifacia*	ms3793	MG683104	MG683591	MG683742
Bactrocera	*nigrofemoralis*	ms7296	MG683181	n.a.*	MG683821
Bactrocera	*nigrotibialis*	ms1034	MG683034	MG683524	MG683680
Bactrocera	*occipitalis*	ms4584	MG683142	MG683630	MG683781
Bactrocera	*oleae*	ms1388	MG683053	MG683538	MG683694
Bactrocera	*quadrata*	ms4539	MG683136	MG683623	MG683774
Bactrocera	*raiensis*	ms1331	MG683050	KT594844	KT594804
Bactrocera	*thailandica*	ms1050	MG683036	MG683526	MG683682
Bactrocera	*tryoni*	ms4504	MG683133	MG683620	MG683771
Bactrocera	*tuberculata*	ms1084	MG683037	MG683527	MG683683
Bactrocera	*umbrosa*	ms1004	MG683031	MG683521	MG683677
Bactrocera	*xanthodes*	ms1426	MG683057	MG683543	MG683698
Bactrocera	*zonata*	ms3741	MG683101	MG683589	MG683740
Ceratitis	*capitata*	ms3495	MG683094	MG683583	MG683733
Dacus	*ciliatus*	ms1572	MG683068	MG683557	MG683707
Zeugodacus	*cucurbitae*	ms1000	MG683030	MG683520	MG683676

* n.a. = data not available

Table S2. Fruit fly samples from olive (*Olea europaea*) investigated in this study and Genbank accession numbers of marker sequences determined.

Genus	Species	Specimen-voucher	Geographic Origin	COI	CAD1	EF1a	Period
Ceratitis	*capitata*	GMF1	Giza; Egypt	MZ350650	MZ358323	MZ358342	MZ358362
Ceratitis	*capitata*	GMF2	Giza; Egypt	MZ350651	n.d.*	MZ358343	MZ358363
Ceratitis	*capitata*	GMM1	Giza; Egypt	MZ350652	MZ358324	MZ358344	MZ358364
Ceratitis	*capitata*	GMM2	Giza; Egypt	MZ350653	MZ358325	MZ358345	MZ358365
Bactrocera	*zonata*	GPF1	Giza; Egypt	MZ350634	MZ358326	MZ358346	MZ358366
Bactrocera	*zonata*	GPF2	Giza; Egypt	MZ350635	MZ358327	MZ358347	MZ358367
Bactrocera	*zonata*	GPF3	Giza; Egypt	MZ350636	MZ358328	MZ358348	MZ358368
Bactrocera	*zonata*	GPM1	Giza; Egypt	MZ350637	MZ358329	MZ358349	MZ358369
Bactrocera	*zonata*	GPM2	Giza; Egypt	MZ350638	MZ358330	MZ358350	MZ358370
Bactrocera	*zonata*	GPM3	Giza; Egypt	MZ350639	MZ358331	MZ358351	MZ358371
Bactrocera	*oleae*	IOF1	Ismailia; Egypt	MZ350646	MZ358332	MZ358352	MZ358372
Bactrocera	*oleae*	IOF2	Ismailia; Egypt	MZ350647	MZ358333	MZ358353	MZ358373
Bactrocera	*oleae*	IOM1	Ismailia; Egypt	MZ350648	MZ358334	MZ358354	MZ358374
Bactrocera	*oleae*	IOM2	Ismailia; Egypt	MZ350649	MZ358335	MZ358355	MZ358375
Bactrocera	*zonata*	IPF1	Ismailia; Egypt	MZ350640	MZ358336	MZ358356	MZ358376
Bactrocera	*zonata*	IPF2	Ismailia; Egypt	MZ350641	MZ358337	MZ358357	MZ358377
Bactrocera	*zonata*	IPF3	Ismailia; Egypt	MZ350642	MZ358338	MZ358358	MZ358378
Bactrocera	*zonata*	IPM1	Ismailia; Egypt	MZ350643	MZ358339	MZ358359	MZ358379
Bactrocera	*zonata*	IPM2	Ismailia; Egypt	MZ350644	MZ358340	MZ358360	MZ358380
Bactrocera	*zonata*	IPM3	Ismailia; Egypt	MZ350645	MZ358341	MZ358361	MZ358381

* n.d. = not determined

Table S3. Single Nucleotide Polymorphisms (SNPs) identified in COI haplotypes of *B. zonata* from olive.

**COI haplotype**	**A**	**B**	**C**	**D**	**E**	**F**	**G**
**Position in COI sequence (573 bp)**	**Nucleotides (deviations from haplotype A are marked yellow)**						
60	T	T	T	T	T	T	C
96	C	C	C	C	C	T	C
126	A	A	A	A	G	A	A
165	C	C	C	T	C	C	C
204	A	A	G	A	A	A	A
255	C	C	C	T	T	C	C
258	A	A	A	G	A	A	A
289	C	C	C	C	C	T	C
309	C	C	C	T	C	C	C
339	C	C	C	C	C	T	C
348	C	C	C	C	C	T	C
370	C	C	C	C	C	T	C
405	T	T	T	C	C	T	T
408	A	A	A	G	A	A	A
438	A	A	A	A	A	A	G
442	C	C	C	C	C	T	C
444	C	C	C	C	C	A	C
481	C	C	C	C	C	T	C
490	T	T	C	T	T	T	T
492	A	G	G	A	A	G	G
504	G	A	G	A	A	A	G
516	T	T	T	T	T	A	T
558	C	C	C	C	T	n.d.*	C
**Fruit fly samples**	IPF3	GPM1<br>GPM3	IPF2	GPF1<br>GPM2<br>IPM2	GPF2<br>GPF3<br>IPF1	IPM3	IPM1

* n.d. = not determined
